# Penetrating Orbital Injury: A Narrative Review for Emergency Clinicians

**DOI:** 10.3390/healthcare13020098

**Published:** 2025-01-07

**Authors:** Florence Pirlet, Julien Flament

**Affiliations:** Emergency Department, CHU UCL Namur, 5530 Yvoir, Belgium; florence.pirlet@student.uclouvain.be

**Keywords:** penetrating orbital injury, orbital rim, occult injury, eyelid injury, traumatology

## Abstract

Penetrating orbit injury is a rare but complex and life-threatening occurrence that may easily be overlooked. Management in the emergency department requires an early multidisciplinary approach but still lacks standard guidelines. This narrative review aims to provide a systematic approach to the management of penetrating orbital injuries for emergency clinicians. Mortality and morbidity are significant due to the orbit’s proximity to numerous anatomical structures. Complications may be infectious, ocular, or cerebro-vascular. Their incidence depends on the mechanism of injury, entry point, and object’s characteristics such as its shape, composition, and velocity. Non-occult cases are often associated with medial orbital rim or medial eyelid penetration, whereas occult cases mainly present with trivial lateral eyelid injury and could be overlooked. Radiological workup consists of computed tomography or magnetic resonance according to the object’s composition. Treatment in the emergency department focuses on initial resuscitation, broad-spectrum antibiotics, and tetanus immunization. Subsequently, early removal of the foreign object in a controlled environment and other specific treatments must be discussed with a multidisciplinary team. Penetrating orbital injury is an uncommon but complex type of head trauma that may be challenging to diagnose. Systematic and multidisciplinary management in the emergency department is crucial to improve overall prognosis.

## 1. Introduction

Penetrating orbital injuries (POIs) are rare but carry a significant risk of morbidity and mortality due to the orbit’s proximity to critical anatomical structures, including the brain, cranial nerves, and major blood vessels. POIs can manifest as either non-occult, with overt clinical signs, or occult, where clinical manifestations are initially minimal, complicating diagnosis. Given the complexities associated with POIs, early and accurate multidisciplinary management is critical, involving emergency physicians, ophthalmologists, neurosurgeons, and radiologists [1,2]. Despite numerous case reports in the literature, there are no standardized guidelines for the optimal management of POIs. This narrative review seeks to provide a systematic approach for emergency clinicians, drawing from the literature and case studies to guide diagnosis and treatment.

## 2. Methods

The authors searched PubMed and Google Scholar for articles using the keyword and medical subject heading “Penetrating orbital injury” OR “Transorbital injury”. Authors included case reports, case series, and reviews of the literature. The literature search was restricted to studies and resources published in English. All of the resources were reviewed and were selected with a focus on emergency-medicine-relevant articles. A total of 29 resources were selected for inclusion in this review.

## 3. Results and Discussion

### 3.1. Epidemiology, Morbidity, and Mortality

**Figure 1:**
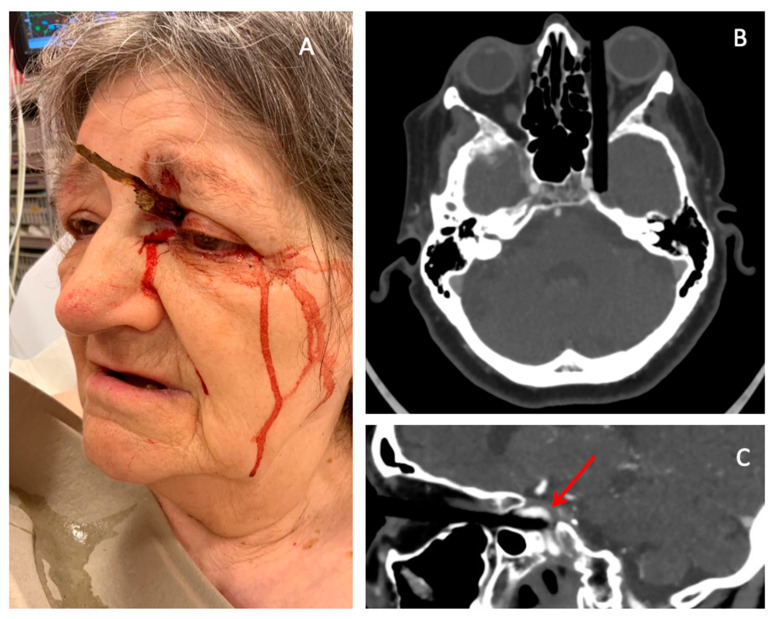
Example of a penetrating orbital injury caused by a tree branch. (**A**) Picture of the patient at presentation in the emergency room. (**B**) Transversal computed tomography view of the patient’s skull. (**C**) Sagittal view showing the tree branch located between the intracavernous segment of the internal carotid artery and the temporal lobe (Arrow). Credits Flament, J.; Moraine, A [8].

POIs account for 30–50% of all orbital injuries and represent 24% of penetrating head traumas in adults and 45% in children, amounting to approximately 0.4% of all head injuries [3,4,5,6]. Common causes include assaults, self-inflicted injuries, motor vehicle accidents, hobbies, and overall accidents involving sharp objects such as windshields, pens, needles, and other trivial objects [2,4,7]. For example, Flament and al reported a case of POI caused by a tree branch following a fall (Figure 1) [8]. The incidence varies with age, sex, and social status, with the peak incidence occurring in children aged 3–9 years [9,10]. Males are four times more likely to sustain POIs than females, although this difference decreases in the elderly due to occupational patterns [9,10].

Historically, POIs had high mortality rates, with a reported 12.5% mortality rate in orbito-cranial wounds during World War II [11]. However, since the advent of antibiotics and advances in imaging and surgical techniques, survival rates have improved significantly [9]. Despite these advances, the prognosis of POIs still largely depends on the initial neurovascular damage and the risk of secondary complications [6].

### 3.2. Mechanism of Injury

**Figure 2:**
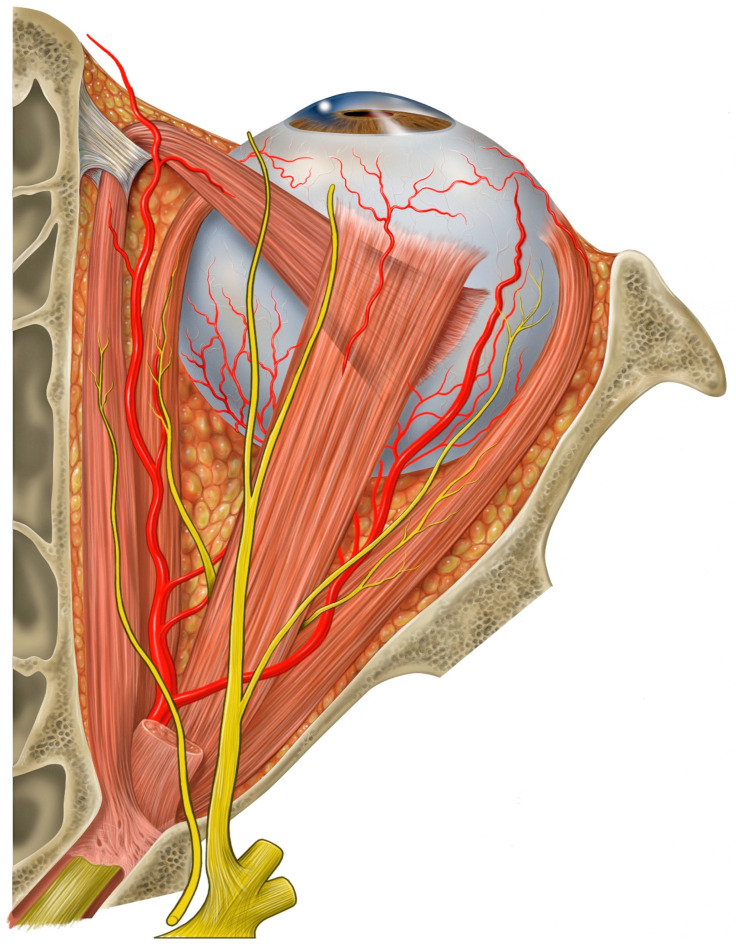
Illustration of the superior view of the orbit showing its pyramidal shape and neurovascular structures. Credits: Patrick J. Lynch, medical illustrator [12].

**Figure 3:**
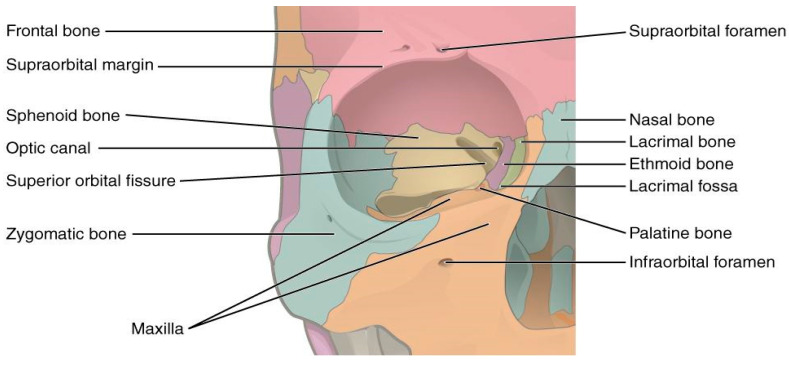
Illustration of the bone structures forming the orbit. Credits: OpenStax College [13].

The anatomy of the orbit, with its thin bony walls and pyramidal shape, makes it vulnerable to penetrating injuries that can affect not only the eye but also adjacent structures such as the brain, cranial nerves, and major blood vessels (Figure 2 and Figure 3) [12,13]. The velocity of the penetrating object plays a crucial role in determining the extent of the damage [2]:

- Missile injuries: Objects like bullets, traveling at velocities greater than 100 m per second, inflict damage primarily through high-energy transfer, causing extensive tissue destruction along their path [2,9,14].
- Non-missile injuries: Objects with lower velocity (<100 m per second), such as chopsticks, branches, or pens, typically result in localized tissue laceration due to the pyramidal configuration of the orbit, which directs the object towards the orbital apex. In civilian settings, most POIs result from low-velocity injuries [2,9,15,16].

### 3.3. Complications

Complications arising from POIs are classified into ocular, infectious, cerebral, and vascular categories [2,6]. The material, size, and velocity of the object, as well as the structures involved, determine the severity and type of complication.

#### 3.3.1. Ocular Complications

Ocular complications from POIs are particularly concerning due to their potential to cause permanent vision loss. These injuries often result from high-velocity impacts but can occur with lower-velocity objects as well. In some cases, globe injuries may be absent due to the globe’s mobility, but the surrounding orbital structures can still be severely damaged [3,17,18]. Common ocular complications include:

- Open globe injuries, which significantly increase the risk of permanent vision loss [17,18].
- Intraocular foreign bodies (IOFBs), which can cause retinal detachment, vitreous hemorrhage, and secondary infections such as endophthalmitis [1,10].
- Optic neuropathy secondary to a direct trauma or compression of the optic nerve, which may cause permanent vision loss; urgent optic nerve decompression may be required [18,19].
- Ophtalmoplegia which can cause diplopia [3,8].
- Hyphema which may elevate intraocular pressure and lead to secondary glaucoma if not managed [10].

#### 3.3.2. Infectious Complications

Infections pose a significant risk, particularly with retained foreign objects. Wooden foreign bodies carry a higher risk of infection compared to metallic ones [15,20]. Infectious complications include:

- Orbital cellulitis, characterized by pain, swelling, proptosis, and fever; orbital cellulitis may progress to life-threatening conditions like meningitis, cavernous sinus thrombosis, or brain abscess if untreated [2,3,9,17,19].
- Endophthalmitis may result in blindness [1,10].
- Meningitis and brain abscess: the proximity of the orbit to the brain makes POIs prone to intracranial infections like meningitis or brain abscess, manifesting as fever, headache, and altered mental status [9,17,21].

#### 3.3.3. Cerebral Complications

POIs that involve the superior orbital rim or medial rim can affect the brain, resulting in cerebral contusions, hematomas, or cerebrospinal fluid (CSF) leaks. Key cerebral complications include:

- Cerebral contusion or brain edema, which can cause intracranial pressure (ICP) elevation, increasing the risk of herniation [9,17].
- Subdural and epidural hematomas, which can lead to significant neurological impairment or seizures if left untreated [2,9,17,21,22].
- Cerebrospinal fluid (CSF) leak may be present when the dura mater is breached. CSF may leak from the nose or superior eyelid, increasing the risk of infection [9,12,19].
- Subarachnoid and epidural hemorrhage, or cerebrospinal fluid (CSF) leaks [17,19].

#### 3.3.4. Vascular Complications

Vascular injuries pose the highest immediate risk due to the potential for life-threatening hemorrhage or aneurysm formation. These include:

- Carotid-cavernous fistula, which can lead to symptoms such as pulsatile exophthalmos and eye pain, requiring angiographic confirmation and endovascular repair [17,19,23].
- Traumatic pseudoaneurysm is at risk of later rupture [17,23,24].
- Subarachnoid and epidural hemorrhage can lead to rapid neurological deterioration, necessitating urgent surgical intervention [2,17,19].

### 3.4. Patterns of Injury

Penetrating orbital injuries (POIs) present diverse trajectories and injury patterns depending on the entry point and the velocity of the object. These injuries are classified as either occult (without clinical manifestations) or non-occult (with clinical manifestations) and can significantly vary in terms of clinical presentation, morbidity, and mortality. The direction and depth of the penetrating object determine the extent of injury, with serious complications often resulting from damage to neurovascular structures, intracranial bleeding, or orbital fractures [15,17,19].

Turbin et al. categorized injuries based on entry zones into the superior, inferior, lateral, and medial orbital rims, with different underlying structures involved in each case [17]. Table 1 summarizes patterns of injury with frequent associated complications.

#### 3.4.1. Superior Rim of the Orbit

The superior rim is often involved in POIs resulting from falls, with the frontal bone’s thinness making the frontal lobe particularly vulnerable [15,17]. Injuries in this zone may manifest as frontal lobe contusions or cerebrospinal fluid (CSF) leaks due to penetration of the orbital roof. These injuries are frequently occult, with subtle external signs like minor lid lacerations [17]. Leaking clear fluid or subtle edema may be the only clinical indication of deeper injury, as documented in cases involving toddlers [15,17].

#### 3.4.2. Inferior Rim of the Orbit

Inferior orbital injuries are less common but typically result from high-velocity traumas, such as stabbings or assaults. The orbital floor’s proximity to the maxillary sinus means objects penetrating the inferior rim may traverse the maxillary sinus or even the palate [15,17,22]. While injuries here can often involve lacerations to structures like the infraorbital nerve, direct injury to the globe or permanent diplopia is less frequent.

#### 3.4.3. Lateral Rim of the Orbit

Penetration via the lateral rim is less commonly documented in the literature, possibly due to the anatomical angulation of the lateral orbital wall [15,17]. Objects that would impact this region often glance off to either the medial aspect or miss penetrating deeply into the orbital cavity. However, when injuries occur, they tend to avoid direct damage to the neurovascular structures due to the greater thickness of the lateral orbital bone [15].

#### 3.4.4. Medial Rim of the Orbit

Penetration through the medial orbital wall, which is thinner than the lateral counterpart, poses a higher risk of injury to structures such as the ethmoidal sinuses and, in severe cases, the brainstem [15,17]. The proximity of the medial wall to the optic nerve increases the risk of vision loss or optic neuropathy in cases of medial orbital injuries [5,15].

#### 3.4.5. Extraorbital Entry

In some cases, objects may penetrate extraorbitally but passes through the orbit towards deeper structures, such as in impalement injuries. These cases can vary strongly depending on the trajectory of the object [17]. For example, in one documented case, a motor vehicle accident caused a windshield wiper control to pass through multiple facial and orbital structures without directly damaging the globe but resulted in ophthalmoplegia and blindness [18].

### 3.5. Radiological Work-Up

Radiological evaluation is a critical component in diagnosing and managing penetrating orbital injuries (POIs), particularly in occult cases where clinical manifestations are subtle or absent [5,18,21]. Given the complexity and potential severity of POIs, it is essential to combine a thorough clinical examination with a comprehensive radiological assessment at the slightest suspicion of injury (Table 2) [3,17].

#### 3.5.1. Computed Tomography (CT)

Non-contrast CT is widely recognized in the literature as the reference imaging modality for POIs. CT enables clinicians to accurately assess the depth, course, and orientation of the penetrating object, as well as detect fractures and retained foreign bodies [5,6,9,17]. While dense foreign bodies such as glass and metals are easily detected, wooden objects pose a challenge due to their similarity in density to air when dry and soft tissue when wet [9,17,25]. Studies show that up to 42% of non-metallic foreign bodies are missed during initial CT imaging, highlighting the need for alternative imaging in certain cases [17].

#### 3.5.2. Magnetic Resonance Imaging (MRI)

MRI is the preferred imaging modality when organic foreign bodies (e.g., wood) are suspected, offering superior contrast resolution for soft tissue. However, MRI should be avoided if there is a suspicion of metallic foreign bodies, as the magnetic fields may dislodge the object [1,2,5,6,9,17].

#### 3.5.3. Angiography

In cases where vascular injury is suspected, either CTA or MRA is recommended to assess for complications such as carotid-cavernous fistulas, pseudoaneurysms, or other vascular injuries [1,18]. Angiographic evaluation is especially crucial in cases involving fractures of the sphenoid’s greater wing or clinical signs suggesting injury to the middle cranial fossa or cavernous sinus [18].

#### 3.5.4. Ultrasonography

While orbital ultrasonography has limited value in managing POIs, it can be helpful in certain ocular conditions when performed by experienced personnel. However, a negative ultrasound result does not rule out a foreign body, and further imaging is often required [3,24].

#### 3.5.5. Conventional Radiography

Conventional radiography is inadequate for evaluating POIs because it often fails to detect orbital fractures or radiolucent foreign bodies [3,17]. CT or MRI should remain the primary modalities for a thorough assessment of these injuries.

### 3.6. Management and Treatment

Penetrating orbital injuries (POIs) require a multidisciplinary team approach involving emergency physicians, ophthalmologists, neurosurgeons, otorhinolaryngologists, maxillofacial surgeons, and radiologists to ensure optimal care [15]. Early and appropriate management is critical to prevent complications and optimize patient outcomes.

#### 3.6.1. Initial Evaluation and Resuscitation

The importance of a thorough and systematic physical examination cannot be overstated. Emergency physicians should prioritize airway protection and hemodynamic stabilization during the initial stages of management [1,5,18]. In cases where patient agitation compromises further investigation, anesthesia and mechanical ventilation may be required [1,18]. Even if the foreign object has already been removed upon arrival at the emergency department, a complete radiological work-up is still essential to identify and manage underlying neurovascular or neurological injuries. Cases of fatal hemorrhage resulting from foreign object removal outside a controlled environment have been reported [1,15,18].

#### 3.6.2. Prophylactic Antibiotics

Broad-spectrum antibiotic therapy should be initiated upon admission to reduce the risk of infection, especially in cases involving organic foreign bodies [1,4,18]. Antibiotics with good central nervous system penetration (e.g., ceftriaxone, ciprofloxacin, metronidazole) are recommended to protect against infections from pathogens like Staphylococcus, Bacillus, and Clostridium species [4,5,18]. Tetanus prophylaxis should be administered to all patients presenting with soil-contaminated objects or metallic foreign bodies [2,16,26]. Overuse of antibiotics must be monitored closely to avoid toxicity and allergic reactions [16]. Additionally, cultures should be obtained upon foreign body removal, particularly when dealing with organic objects [18].

#### 3.6.3. Surgical Management

Surgical intervention is indicated in cases involving retained foreign bodies, bone fractures, cerebrospinal fluid (CSF) leaks, intracranial hematomas, or vascular injuries [9,15,18]. The primary goals of surgery include neurovascular decompression, direct removal of the foreign object under visualization, debridement, hemostasis, and stabilization of fractures [5,18]. Surgery within 3–12 h of injury is recommended to reduce the risk of complications [18]. The surgical approach must be individualized, depending on the location and trajectory of the foreign body, and discussed among the interdisciplinary team [18]. Endoscopic extraction is a less invasive alternative for medial orbital foreign bodies and has been shown to be effective in reducing complications [27,28]. In certain cases, CT-guided extraction has also proven to be a safe and effective method for the removal of complex foreign bodies [29].

#### 3.6.4. Postoperative Follow-Up

All patients should undergo follow-up imaging after surgery, typically with CT scanning, to ensure proper healing and the absence of retained foreign bodies [5,18]. Patients with injuries to the internal carotid artery or other vascular structures should undergo MRI angiography or CTA within 1–3 months post trauma to detect potential vascular complications such as pseudoaneurysms [18]. Ophthalmologic follow-up is essential, particularly for patients with optic nerve injuries or other sight-threatening complications.

**Figure 4:**
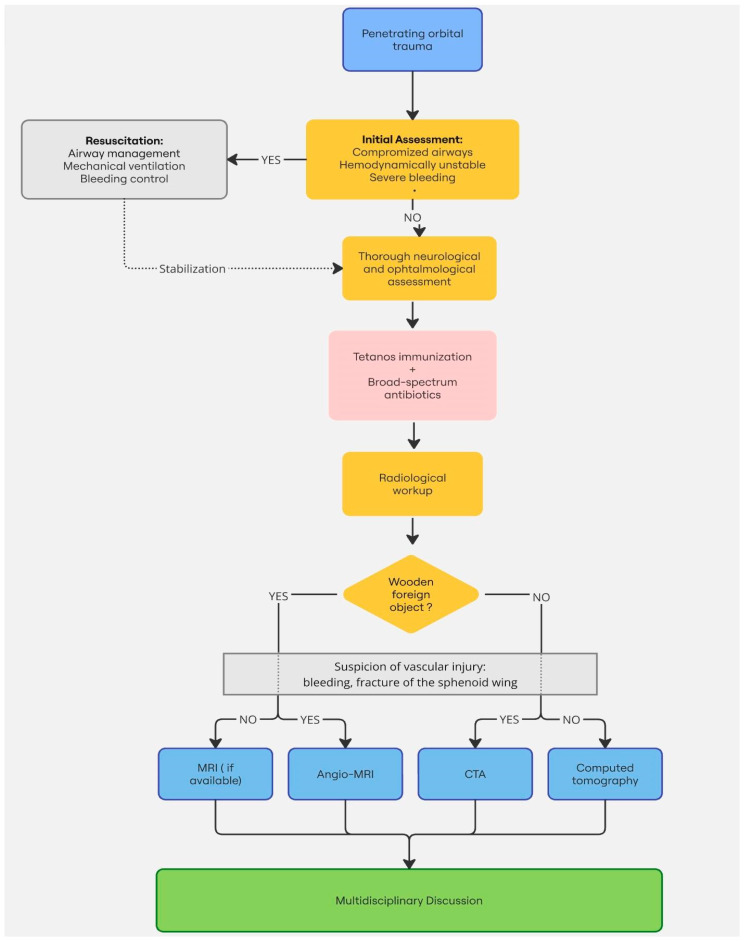
Suggested management algorithm of POIs.

In cases involving optic nerve trauma, the use of high-dose corticosteroids remains controversial and is not routinely recommended due to limited supporting evidence [5,18] (Figure 4).

## 4. Conclusions

Penetrating orbital injuries, though rare, require a systematic and multidisciplinary approach due to their potential for significant morbidity and mortality. Emergency clinicians must maintain a high index of suspicion for POIs, even in cases where clinical signs are not immediately apparent. Prompt imaging and surgical intervention are key to minimizing complications and improving outcomes. Advances in imaging and surgical techniques, particularly endoscopic approaches, have significantly enhanced the management of POIs and offer promising outcomes for patients who receive timely care.

## Figures and Tables

**Table 1:** Summary of patterns of penetrating orbital injury and associated complications.

Zone of Penetration	Involved Structures	Common Trajectories	Underlying Injuries	Clinical Findings	References
Superior Orbital Rim	Frontal bone, orbital roof, frontal lobe	Penetrates orbital roof into frontal lobe	Frontal lobe contusion, cerebrospinal fluid leaks	Lid laceration, clear fluid leakage, frontal lobe oedema	[5,15,17,18,19,22]
Inferior Orbital Rim	Infraorbital nerve, maxillary sinus, palate	Penetrates orbital floor into maxillary sinus and palate	Infraorbital nerve laceration, diplopia, maxillary sinus injury	Numbness, diplopia, infraorbital swelling, palatal perforation	
Lateral Orbital Rim	Zygomatic bone, lateral orbital wall	Penetretes lateral orbital wall without deep penetration	Zygomatic fracture, superficial trauma	Minimal penetration, lateral orbital swelling, often no deep neurovascular injury	
Medial Orbital Rim	Ethmoidal sinuses, optic nerve, brainstem (severe)	Penetrates medial orbital wall into sinuses or deeper	Optic nerve damage, ethmoidal sinus injury, brainstem contusion	Vision loss, optic neuropathy, sinus infection, potentially life-threatening brainstrem comlications	
Extraorbital Entry	Varies (e.g., maxillary sinus, nasal cavity)	Enters via facial structures but involves orbital tissues	Ophtalmoplegia, optic nerve damage, ethmoidal fracture	Blindless, cranial nerve palsies, globe rupture in some cases	

**Table 2:** Imaging modalities for POI and indications.

Imaging Modality	Indication	Advantages	Limitations	References
**Non-Contrast CT**	Initial assessment, detection of fractures, metallic foreign bodies	Quick, widely available, good for fractures and metal	May miss organic materials like wood	[1,2,3,5,6,7,9,17,18,24,25]
**MRI**	Suspected organic foreign bodies, soft tissue assessment	Superior soft tissue contrast, identifies non-metal objects	Contraindicated with metallic foreign bodies	
**CTA/MRA**	Suspected vascular injury (carotid-cavernous fistula, pseudoaneurysm)	Visualizes blood vessels, identifies vascular complications	Limited in acute trauma with metal	
**Orbital Ultrasonography**	Assessment of soft tissue or fluid collection	Non-invasive, real-time imaging, useful for ocular conditions	Limited scope, requires specific expertise	
**Orbital Ultrasonography**	Initial screening in resource-limited settings	Readily available, low cost	Poor sensitivity for radiolucent objects like glass	

## References

[1] Barouj M.D., Tabrizi R., Behnia P., Tabrizi M.A.A., Kheirkhahi M. (2020). Penetrating Orbital Injury; a Case Report and Treatment Algorithm. Arch. Acad. Emerg. Med..

[2] Tabibkhooei A., Aslaninia A., Anousha K. (2019). Childhood Transorbital Skull Base Penetrating Injury: Report of 2 Cases and Review of Literature. World Neurosurg..

[3] Gonullu M.E., Filinte G.T., Cardak N.G.A., Kucuk S., Akoz T. (2016). The Surgical Strategy for the Intraorbital Foreign Bodies. J. Craniofac. Surg..

[4] Rzaev D.A., Danilin V.E., Letyagin G.V., Istomina T.K., Chishchina N.V. (2017). Penetrating orbitocranial injury: A review of the literature and a case report of injury by a watercolor brush in a 3-year-old child. Zh Vopr Neirokhir Im NN Burdenko.

[5] Mzimbiri J.M., Li J., Bajawi M.A., Lan S., Chen F., Liu J. (2016). Orbitocranial Low-Velocity Penetrating Injury: A Personal Experience, Case Series, Review of the literature, and Proposed Management Plan. World Neurosurg..

[6] Agrawal A., Reddy V.U., Kumar S.S., Hegde K.V., Rao G.M. (2016). Transorbital Orbitocranial Penetrating Injury with an Iron Rod. Craniomaxillofac. Trauma Reconstr..

[7] Santander X.A., Revuelta J.M., Cotúa C., Rodriguez B.A., de Leyva Moreno P., Mazzei A.S. (2019). Occult Transorbital Intracranial Injury by Windshield Wiper Handle: Case Report and Review of Literature. World Neurosurg..

[8] Flament J., Moraine A. (2021). Tree Branch through the Orbit into the Skull: A Case Report. Radiol. Case Rep..

[9] Duinen M.T.A. (2000). The Transorbital Intracranial Penetrating Injury.

[10] Négrel A.D., Thylefors B. (1998). The global impact of eye injuries. Ophthalmic Epidemiol..

[11] Webster J.E., Schneider R.C., Lofstrom J.E. (1946). Observations upon the Management of Orbito-Cranial Wounds. J. Neurosurg..

[12] Wikimedia Commons contributors Eye Orbit Anatomy Superior. https://commons.wikimedia.org/wiki/File:Eye_orbit_anatomy_superior.jpg.

[13] Betts J.G., Young K.A., Wise J.A., Johnson E., Poe B., Kruse D.H., Korol O., Johnson J.E., Womble M., DeSaix P. (2013). Anatomy and Physiology.

[14] Awori J., Wilkinson D.A., Gemmete J.J., Thompson B.G., Chaudhary N., Pandey A.S. (2017). Penetrating Head Injury by a Nail Gun: Case Report, Review of the Literature, and Management Considerations. J. Stroke Cerebrovasc. Dis..

[15] Mashriqi F., Iwanaga J., Loukas M., D’Antoni A.V., Tubbs R.S. (2017). Penetrating Orbital Injuries: A Review. Cureus.

[16] Dunya I.M., Rubin P.A., Shore J.W. (1995). Penetrating orbital trauma. Int. Ophthalmol. Clin..

[17] Turbin R.E., Maxwell D.N., Langer P.D., Frohman L.P., Hubbi B., Wolansky L., Mori M. (2006). Patterns of transorbital intracranial injury: A review and comparison of occult and non-occult cases. Surv. Ophthalmol..

[18] Schreckinger M., Orringer D., Thompson B.G., La Marca F., Sagher O. (2011). Transorbital penetrating injury: Case series, review of the literature, and proposed management algorithm. J. Neurosurg..

[19] Ghadersohi S., Ference E.H., Detwiller K., Kern R.C. (2017). Presentation, workup, and management of penetrating transorbital and transnasal injuries: A case report and systematic review. Am. J. Rhinol Allergy.

[20] Li J., Zhou L.P., Jin J., Yuan H.F. (2016). Clinical diagnosis and treatment of intraorbital wooden foreign bodies. Chin. J. Traumatol..

[21] Di Roio C., Jourdan C., Mottolese C., Convert J., Artru F. (2000). Craniocerebral injury resulting from transorbital stick penetration in children. Childs Nerv. Syst..

[22] Subburaman N., Sivabalan K., Ramachandran M., Chandrasekhar D. (2005). Impacted knife injury of the orbit, maxilla and oropharynx. Indian J. Otolaryngol. Head Neck Surg..

[23] Tenenholz T., Baxter A.B., McKhann G.M. (1999). Orbital assault with a pencil: Evaluating vascular injury. AJR Am. J. Roentgenol..

[24] Carothers A. (1978). Orbitofacial wounds and cerebral artery injuries caused by umbrella tips. JAMA.

[25] Kim Y.H., Kim H., Yoon E.S. (2018). Unrecognized intraorbital wooden foreign body. Arch. Craniofac. Surg..

[26] Callison C., Nguyen H. (2024). Tetanus Prophylaxis. StatPearls.

[27] Ildan F., Bagdatoglu H., Boyar B., Doganay M., Çetinalp E., Karadayi A. (1994). The nonsurgical management of a penetrating orbitocranial injury reaching the brain stem: Case report. J. Trauma Acute Care Surg..

[28] Huang Y.T., Kung W.H., Chang C.H., Ku W.N., Tien P.T., Chiang C.C., Tsai Y.Y. (2020). Endoscopy-assisted extraction of orbital and nasal foreign body. Taiwan J. Ophthalmol..

[29] Sollini G., Giorli A., Zoli M., Farneti P., Arena G., Astarita F., Mazzatenta D., Pasquini E. (2024). Endoscopic transnasal approach to remove an intraorbital bullet: Systematic review and case report. Acta. Otorhinolaryngol. Ital..

